# Effect of active lipid‐based coating incorporated with nanoclay and orange peel essential oil on physicochemical properties of *Citrus sinensis*

**DOI:** 10.1002/fsn3.681

**Published:** 2018-07-02

**Authors:** Seyedeh Zahra Nasirifar, Yahya Maghsoudlou, Najme Oliyaei

**Affiliations:** ^1^ Department of Food Science and Technology College of Food Technology University of Agricultural Sciences and Natural Resources Gorgan Iran; ^2^ Department of Food Science and Technology Shiraz University Shiraz Iran

**Keywords:** blood orange, carnauba wax, coating, MMT

## Abstract

The aim of this study was to evaluate the different lipid‐based coating on the physicochemical properties and shelf life of blood orange. In this study, four different carnauba wax coatings formula were used: carnauba wax, carnauba wax incorporated with orange peel essential oil (OPEO) (1%), carnauba wax with montmorillonite nanoclay (MMT) (2%), and carnauba wax combination by OPEO (0.5%) and MMT (1%). Physicochemical properties (total phenol content, antioxidant activity, °Brix, titratable acidity, vitamin C, color, firmness, and pH) of fruits were determined throughout the storage. According to the results, carnauba wax with MMT was better than the other treatments. The highest antioxidant activity was observed in carnauba wax coating containing MMT and total phenol and DDPH gained 733.00 ± 1.204 (mg gallic acid/100 g) and 78.327 ± 0/364%, respectively, at 100th day. Blood orange coated by carnauba wax with MMT had the least of deformation and dissolved solid and the highest acidity rather than other treatments. Moreover, time storage and coating had significant effect on vitamin C content in which maximum and minimum amount was observed in wax coating incorporated by MMT and combination with MMT and OPEO treatments, respectively. Fruits coating with MMT showed better brightness.

## 1 INTRODUCTION

*Citrus sinensis* varieties Moro, also known as blood oranges and typically grown in the Etna volcano region of Sicily (Italy) as well as in Florida (USA), are characterized by their unique flesh and rind color due to red pigments belonging to anthocyanin class (Kelebek, Canbas, & Selli, 2008; Scordino et al., 2015; Selli & Kelebek, 2011). Blood oranges with striking color have significant health‐promoting properties, combining the high content of vitamin C, carotenoids, and fiber of common blond oranges with the health‐promoting properties of anthocyanin pigments (Davies, 2007; De Pascual‐Teresa, Moreno, & García‐Viguera, 2010; Paredes‐López, Cervantes‐Ceja, Vigna‐Pérez, & Hernández‐Pérez, 2010). In addition, their high antioxidant activity is due to their high anthocyanin content (Jayaprakasha & Patil, 2007; Kelebek et al., 2008) which could reduce oxidative stress in diabetic patients (Bonina et al., 2002).

In addition, the extension of the shelf life of food products is critically dependent on three factors including reduction of desiccation, reduction of the physiologic process of maturation and senescence, and reduction in the onset and rate of microbial growth. For this respect, the use of individual coating of fruits and vegetables could be an important approach to minimize or eliminate these problems and preserve the quality and freshness (Forato, de Britto, de Rizzo, Gastaldi, & Assis, 2015; Yaman & Bayoιndιrlι, 2002). The hydrocolloid coating possesses good barrier properties to water loss, desiccation, and gas exchange. In this regard, a wide range of polymers such as proteins, polysaccharides, and lipids, can be used alone or in combinations with the edible coatings formulation (Azeredo, Miranda, Ribeiro, Rosa, & Nascimento, 2012; Bourtoom, 2008; Chiumarelli & Hubinger, 2012; Danalache, Carvalho, Alves, Moldão‐Martins, & Mata, 2016; Guerreiro, Gago, Faleiro, Miguel, & Antunes, 2017; Vargas, Pastor, Chiralt, McClements, & Gonzalez‐Martinez, 2008). Lipids commonly used in the coating formulation are stearic acid, palmitic acid, and some vegetable oils, such as soybean and sunflower (Colla, Do Amaral Sobral, & Menegalli, 2006; Martín‐Belloso, Soliva‐Fortuny, & Baldwin, 2005); however, natural and synthetic waxes showing good gas barrier and better moisture barrier properties than coatings contain only fatty acids (Rojas‐Argudo, del Río, & Pérez‐Gago, 2009; Talens & Krochta, 2005).

Nanocomposite polymers are alternative technologies for improving polymer properties which exhibit increased barrier properties, mechanical strength, and improved heat resistance compared to their neat polymers and conventional composites (Boelter & Brandelli, 2016; Sorrentino, Gorrasi, & Vittoria, 2007). Furthermore, nanosized clays could be used as particle fillers which include the MMTs montmorillonite (MMT) and kaolinite, carbon nanotubes, and graphene nanosheets (Echegoyen, 2015; Shokrieh, Saeedi, & Chitsazzadeh, 2013). There are three types of polymer–clay formations namely (1) tactoid, (2) intercalated, and (3) exfoliated (Ray & Okamoto, 2003) which improve material properties, compare with the matrix polymers alone (Ray and Okamoto, 2003).

However, potential functions and applications of the coatings warrant increased considerations. Extensive research is still needed on the methods of coating formation and improvement of their properties and applications.

The aim of this study was to preserve the physicochemical traits and prolonging the shelf life of blood orange with coating. In this study, we evaluated the effect of carnauba wax alone and carnauba wax incorporated with orange peel essential oil (OPEO), carnauba wax with MMT and carnauba wax combination by orange peel essential oil and MMT and then investigated their physicochemical properties.

## 2 MATERIALS AND METHODS

### 2.1 Materials

*Citrus sinensis* varieties Moro were collected from mazandaran's garden (south of Iran). MMT supplied by American Southern‐Clay company (commercial name: Cloisite Na+, organic modifier: 2M2HT (dimethyl, dehydrogenated tallow, quaternary ammonium), modifier concentration: 125 (meq/100), X‐ray dispersion: 31.5 (d001 A°)). Methanol, Folin–Ciocalteu reagent, sodium carbonate, gallic acid, DPPH reagents, sodium hydroxide, represents the NBS, acetic acid, phenolphthalein, potassium iodide, starch were purchased from Sigma Chemical Co.

### 2.2 Fruits coating

In this study, different wax coatings, including carnauba wax +2% MMT, carnauba wax +1% OPEO, carnauba wax +1% MMT + 0.5% OPEO, and carnauba wax alone, were evaluated. Each formulation was sprayed on *Citrus sinensis* fruits and then stored at 7°C and 85% RH for 100 days. The quality properties of fruits were measured every 20 days.

### 2.3 Total phenol content

Total phenols were determined by the method of McDonald, Prenzler, Antolovich, and Robards (2001) using the Folin–Ciocalteu reagent. An aliquot (20 ml) of orange juice was added to 1.16 ml of distilled water, followed by 100 ml of Folin–Ciocalteu solution. After 5 min, 300 ml of 20% sodium carbonate was added and the contents of the tube were thoroughly mixed before being incubated in a water bath (40°C) for 30 min. The tube was allowed to cool in the dark. The absorbance of samples was read at 760 nm using gallic acid as standard. Results were expressed in mg gallic acid per 100 g dry weight.

### 2.4 DPPH radical assay

An ethanol solution of 0.1 mmol/L DPPH was added to 1 ml orange juice and after mixing for 10 s, the solution was stored in dark room for 30 min. Afterward, the absorbance of resulting solution was measured at 515 nm by spectrometer (T80 + UV/VIS, PG Instrument Ltd, America). DPPH solution was used as control which had maximum absorbance (Koleva, van Beek, Linssen, Groot, & Evstatieva, 2002). The activity was measured according to the following equation:

$$\%\text{DPPH}=\frac{\text{control absorbance}-\text{sample absorbance}}{\text{Control absorbance}}\times 100$$

### 2.5 Total soluble solids

Total dissolved solids of supernatant of orange juice from centrifuging (3,500 rpm, 10 min) were measured by refractometer (ABB modle, CETi company, Belgium) (Chien, Sheu, & Lin, 2007).

### 2.6 Titratable acidity

Titratable acidity was determined by titration of orange juice with 0.1 N NaOH solution and expressed as lactic acid percentage (AOAC, 2005). Phenolphthalein was used as detergent.

### 2.7 Vitamin C

A modified method of Egan, Kirk, and Sawyer (1981) was used for the measurement of vitamin C from orange juice. 5 ml orange juice was added to 60 ml distilled water and then was homogenized with 4% potassium iodide, 0.4 ml acetic acid (10%), and 0.3 ml starch (1% concentration). The mixture was then titrated by NBS solution.

### 2.8 Color

To evaluate the color of oranges, samples were placed in a dark box white D65 lamp. The photography was carried out by a digital camera (Canon, 16 Mega Pixels) with perpendicular distance of 30 cm. The color components (L*, a*, and b*) were calculated according to the method described by Yaman and Bayoιndιrlι (2002), using image j software.

### 2.9 Firmness

Three blood oranges from each coating formulation were used for firmness analysis. Firmness was measured with instron SANTAM and evaluated based on deformation percentage (Valencia‐Chamorro, Pérez‐Gago, del Río, & Palou, 2009). Fruits were pressed on plane approaching at speed of 5 mm/min. Deformation was showed after 10 N loading and results were expressed as the percentage change compared to the initial diameter.

### 2.10 pH

PH was measured using digital pH meter (UB‐10, DENVER INSTRUMENT company, America) according to AOAC (2005).

### 2.11 Statistical analysis

The data obtained from experiments were analyzed using completely randomized factorial design by SAS software (SAS Institute, Cary, NC, USA), and the differences between means were evaluated using Duncan's multiple range test. All experiments were done in triplicate.

## 3 RESULTS

### 3.1 Total phenol content

**Figure 1:**
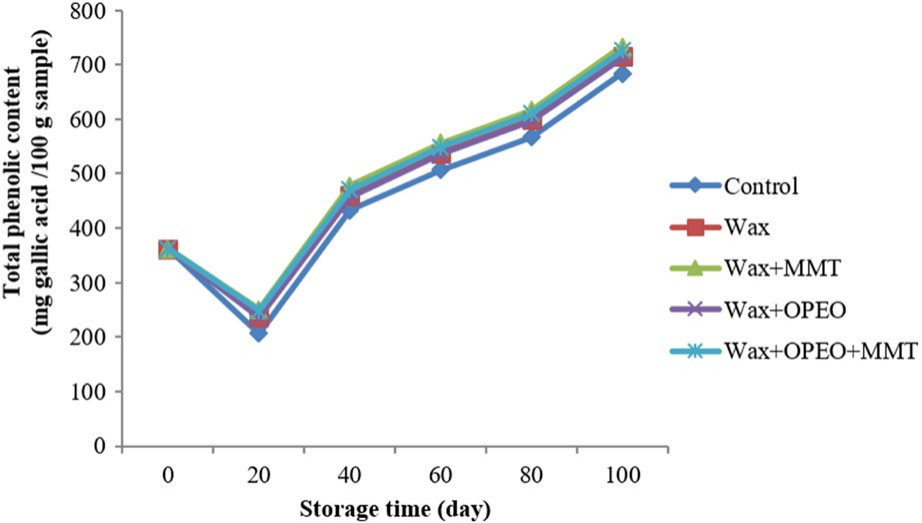
Effect of storage and coating on total phenol

Figure 1 shows the total phenol content of both uncoated sample and those coated with the different formulations throughout the storage time. As can be seen, time and coating had a significant effect on total phenol (*p* < 0.05). Total phenol sharply decreased significantly during the first 20 storage days for all treatments, regardless of the coating treatment, and a progressive trend occurred afterward. On the other hand, 362.47 mg gallic acid/100 g sample reached to 714.20 mg gallic acid/100 g sample (Table 1). Moreover, MMT and MMT + OPEO, OPEO, and wax had no differences (*p* < 0.05); however, significant difference was observed with control (*p* > 0.05).

**Table 1:** Antioxidant activity in different coatings and storage (day)

Storage time (day)	Coating				
	Control	Wax coating	Wax + MMT	Wax + OPEO	Wax + OPEO + MMT
20	59.11 ± 0.43^m^	61.03 ± 0.00 ^l^	62.42 ± 0.45^jk^	59.34 ± 0.43^m^	62.11 ± 0.28^lk^
40	62.83 ± 0.39^j^	65.31 ± 1.30^i^	70.47 ± 0.44^ef^	64.00 ± 0.39^g^	69.40 ± 0.03^gf^
60	63.43 ± 0.23^h^	69.03 ± 1.00 ^g^	75.02 ± 0.43^d^	67.01 ± 0.23^h^	74.28 ± 1.03^d^
80	68.00 ± 0.35^g^	71.00 ± 0.22^e^	78.08 ± 0.44^ab^	69.00 ± 0.35^g^	77.10 ± 0.97^c^
100	68.62 ± 0.51^g^	70.58 ± 0.33^e^	78.33 ± 0.36^a^	69.05 ± 0.51^gf^	77.19 ± 1.00^bc^

Note

Data are expressed as mean ± *SD* (*n* = 3). Different letters in each column indicate that the means are significantly different (*p* < 0.05).

### 3.2 DPPH radical assay

The antioxidant activity of samples was determined by the DPPH radical scavenging assay. DPPH scavenging activity assay is widely used to evaluate the ability of compounds to scavenge free radicals or donate hydrogen, and determine the antioxidant activity in foods (Bidchol, Wilfred, Abhijna, & Harish, 2011). According to Table 2, time and coating had significant effect on inhibition of DPPH radicals (*p* > 0.5). The antioxidant activity of samples increased during storage (*p* < 0.05), then remained relatively constant at the end of the storage. On the other hand, the initial DPPH from 59.39 reached to 72.72%. The best antioxidant activity of juice was observed for wax + MMT‐coated fruits, whereas the lowest was attributed to control, however, had no significant difference with wax + OPEO treatment (*p* < 0.05). Also there were no differences between 80th and 100th day (Table 2).

**Table 2:** Vitamin C content in different coating and storage time (day)

Storage time (day)	Coating				
	Control	Wax coating	Wax + MMT	Wax + OPEO	Wax + OPEO + MMT
20	57.85 ± 0.20^a^	58.150 ± 0.41^a^	58.78 ± 0.04^a^	58.29 ± 0.29^a^	59.12 ± 0.09^a^
40	55.34 ± 0.01^bcd^	55.77 ± 0.13^bcd^	58.78 ± 0.54^a^	56.51 ± 0.14^b^	58.89 ± 0.65^a^
60	53.25 ± 0.28^g^	53.76 ± 0.58^efg^	55.84 ± 1.35^bcd^	54.43 ± 0.63^defg^	56.17 ± 1.38^bc^
80	49.03 ± 0.32^ij^	49.74 ± 0.21^hi^	54.78 ± 2.02^cdef^	50.54 ± 0.08^h^	55.17 ± 2.12^bcde^
100	46.26 ± 0.37^l^	46.74 ± 0.51^kl^	53.22 ± 0.56 ^g^	47.76 ± 0.34^kj^	53.62 ± 0.63^fg^

Note

Data are expressed as mean ± *SD* (*n* = 3). Different letters in each column indicate that the means are significantly different (*p* < 0.05).

### 3.3 Total soluble solids (°Brix)

**Figure 2:**
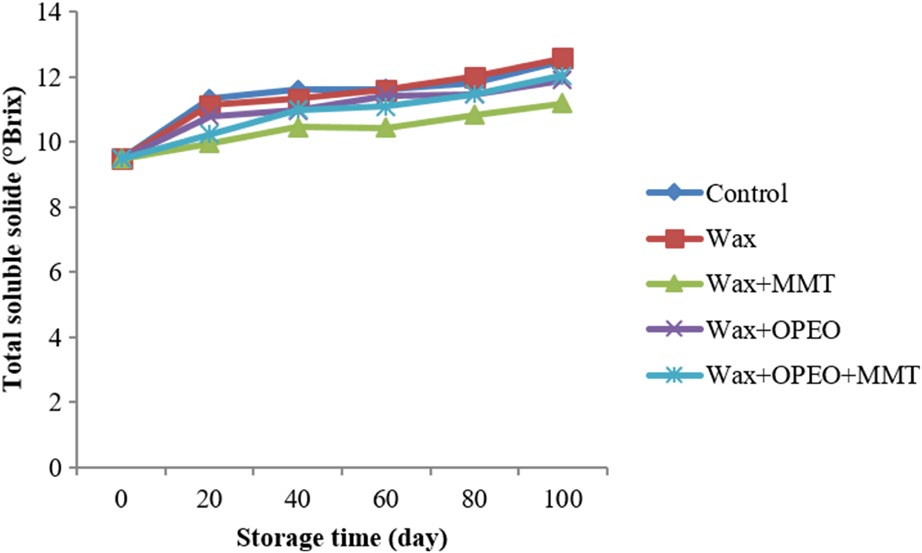
Effect of storage and coating on total dissolved solid of orange

According to analysis of variance (Figure 2), the effects of time and coating had significant effect on soluble solids; however, there was no significant interaction between time and coating (*p* < 0.05). Moreover, the results showed that total soluble solids gradually increased during storage, that is reached (12.4) after 100‐day storage. Also the highest one was observed in control coating which had no significant difference with Wax treatment (*p* < 0.05) and the lowest one was related to MMT + wax coating. The interaction effect of coating and time indicates maximum and minimum, respectively, were relevance to wax coating and MMT + wax at 100th day which had no difference with MMT + OPEO and OPEO (*p* < 0.05).

### 3.4 Vitamin C

Table 3 shows the results of vitamin C. The results indicated that the time, coating, and their interaction had effective influence on vitamin C (*p* > 0.05) and in general a decline in the amount of vitamin C for all coated and uncoated fruits during storage was observed. The initial amount of vitamin C was 61.34 mg/100 g; however, after 100 days of storage, it reached to 49.52 mg/100 g. Moreover, the highest amount of vitamin C is obtained from clay treatment and clay + OPEO treatments although control sample had the lowest one (Table 3). Moreover, the analysis of time and coating interaction effect showed high amount of vitamin C at 100th day in MMT + OPEO‐coated fruits which was the same as MMT treatment and the lowest one was related to control which had no difference with wax coating alone (*p* < 0.05).

**Table 3:** Effect of storage time and coating on pH

Storage time (day)	Coating				
	Control	Wax coating	Wax + MMT	Wax + OPEO	Wax+ OPEO + MMT
20	3.27 ± 0.06^defg^	3.16 ± 0.01^fgh^	3.06 ± 0.01^gh^	3.08 ± 0.01^gh^	3.03 ± 0.06 ^h^
40	3.23 ± 0.16^defgh^	3.15 ± 0.21^fgh^	3.18 ± 0.11^fgh^	3.22 ± 0.14^defgh^	3.18 ± 0.03^fgh^
60	3.32 ± 0.13^cdef^	3.20 ± 0.15^efgh^	3.29 ± 0.17^def^	3.26 ± 0.17^defgh^	3.18 ± 0.3^fgh^
80	3.42 ± 0.03^bcde^	3.43 ± 0.06^bcd^	3.45 ± 0.15^abcd^	3.53 ± 0.17^abc^	3.41 ± 0.14^bcde^
100	3.58 ± 0.03^ab^	3.54 ± 0.05^ab^	3.60 ± 0.13^ab^	3.66 ± 0.18^a^	3.56 ± 0.13^ab^

Note

Data are expressed as mean ± *SD* (*n* = 3). Different letters in each column indicate that the means are significantly different (*p* < 0.05).

### 3.5 Titratable acidity

**Figure 3:**
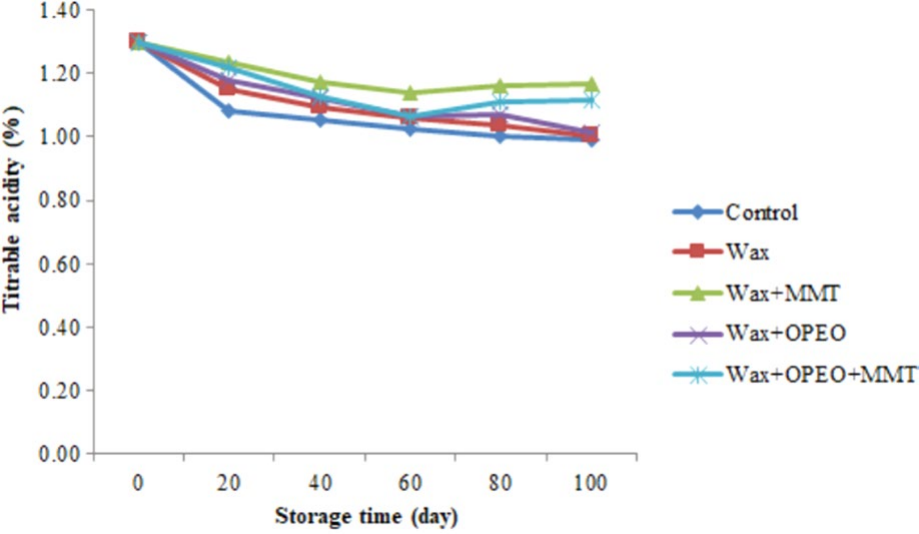
Effect of storage time and coating on titratable acidity

According to Figure 3, coating beneficially influenced titratable acidity of blood orang after 100 days of storage (*p* < 0.05). However, acidity increased gradually in all samples and reached to 1.06% by coating. The MMT + wax‐treated fruits had higher acidity rather than others. Thereafter, wax + OPEO + MMT, wax + OPEO, and wax, respectively, showed higher acidity compared with control.

### 3.6 Firmness

**Figure 4:**
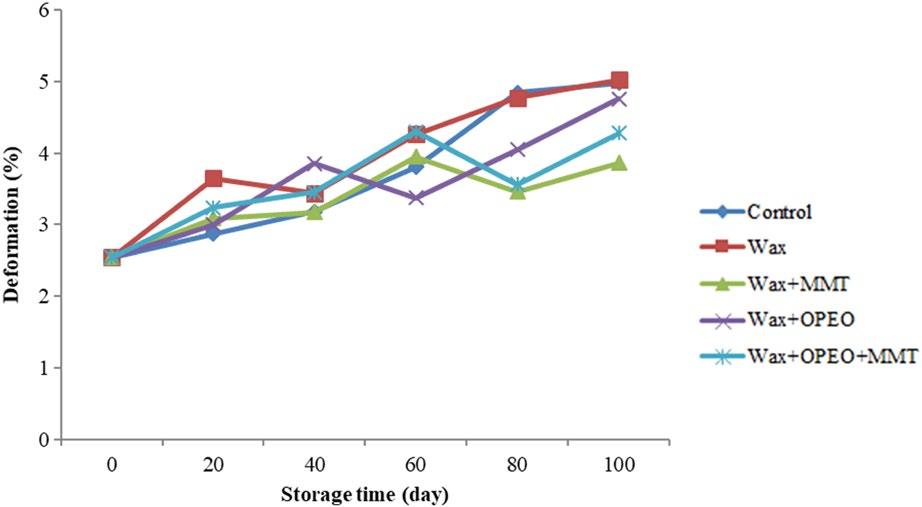
Effect of storage time and coating on firmness

Firmness is one of the most critical quality attributes influencing consumer appeal and marketing of fresh fruit. Figure 4 shows the firmness of blood oranges treated with different carnauba wax coatings. According to the results, deformation of all samples increased throughout the whole storage period and types of coating had significant effect on firmness (*p* < 0.05). The control and wax coated of oranges had the similar deformation changes at 100th day although fruits treated by coatings containing the MMT exhibited better firmness attribute. Moreover, deformation percentage from 2.55% at the beginning reached to 4.58% during the 100 days storage. The best result of firmness was related to wax+MMT treatment (*p* < 0.05).

### 3.7 Color

**Figure 5:**
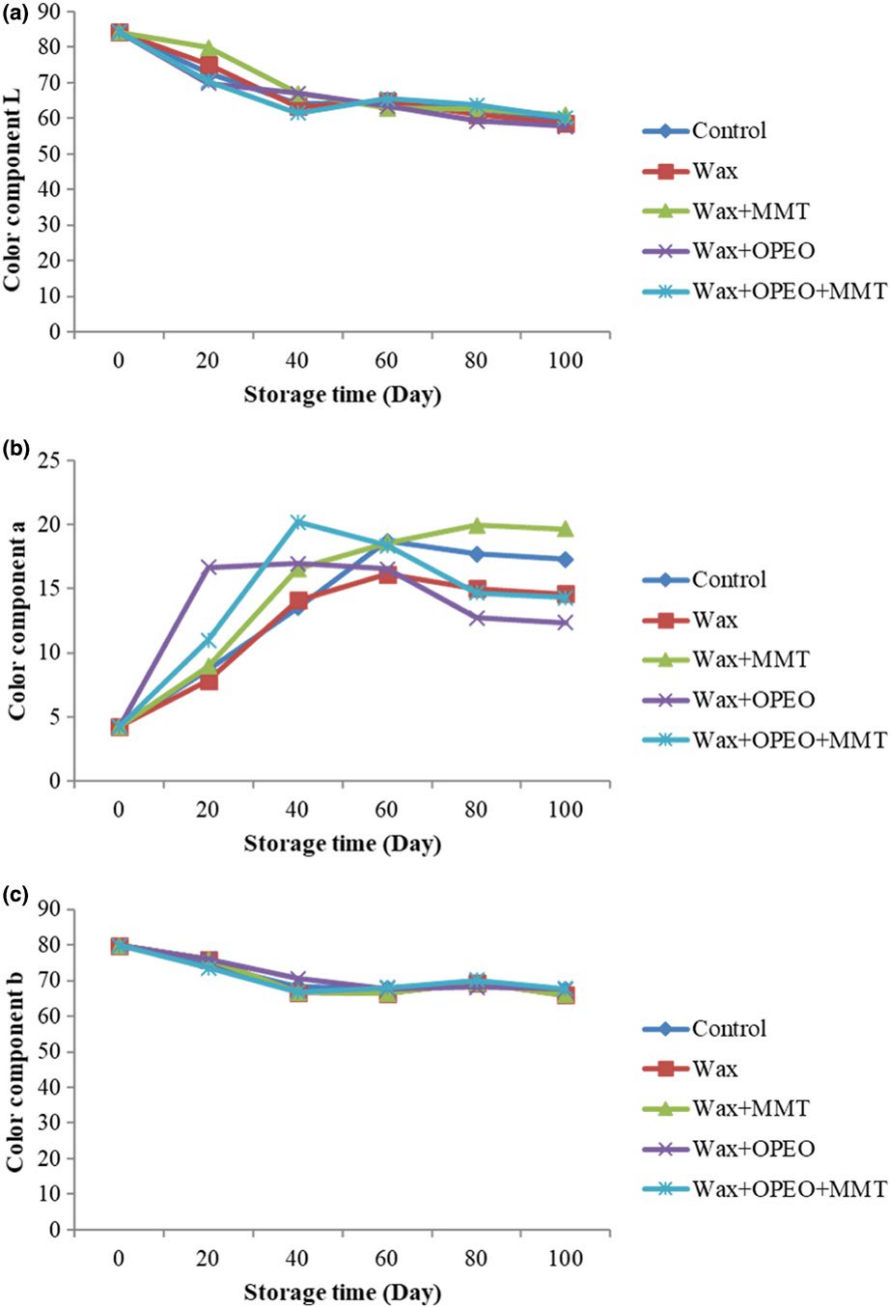
Effect of storage time and coating on (a) color component “L,” (b) color component “a,” and (c) color component “b”

The color parameters of fruits coated are illustrated in Figure 5. The results showed that the color of samples changed during the storage period (*p* < 0.05). According to Figure 5a, it can be noticed that component “L” decreased in all treatments during the storage time (*p* < 0.05) and changed approximately from 83.31 at the beginning to 59.43 at 100th day. Moreover, oranges coated with carnauba wax+MMT had higher “L” parameter and lower one was related to those treated with OPEO. However, no significant differences were observed between control, MMT + OPEO, and wax treatment.

“a” value varied significantly during the storage and after the storage, the intensity of redness decreased (*p* < 0.05). Figure 5b, illustrated that “a” value increased at the middle of the storage, but afterward, a sharp reduction of redness was observed in fruit coated containing OPEO, whereas control and wax coating fruits with slight reduction remained unchanged at 100th day. Blood oranges coated with wax + MMT showed the highest value of redness.

In contrast, component “b” had descending trend during the time (Figure 5c) and reached 66.68 at the end of storage and again MMT had the maximum amount which had no significant difference with control and MMT + OPEO (*p* < 0.05) and OPEO treatment had the minimum one.

### 3.8 pH

The pH values showed significant changes during the storage although there were no differences among the samples. According to Table 3 the pH of samples increased during the storage and reached to 3.54 at the end of the storage in control, whereas the mean pH values for the wax + OPEO coating ranged from 3 to 3.69 (*p* < 0.05). Moreover, on comparing different coating treatment revealed that wax + POEO had the maximum (3.36) and wax + OPEO + MMT had the lowest (3.27) pH.

## 4 DISCUSSION

Citrus fruits are non‐climacteric; hence, their respiration rate and ethylene production do not increase remarkably during the ripening as in climacteric fruits. However, they suffer from some physiologic postharvest disorders such as water loss, peel pitting, and chilling injury at unappropriated storing conditions (Lester & Hodges, 2008; Wardowski, Hall, & Grierson, 2006). Fruit coating is a common approach to reduce the weight loss and improve appearance which is typically based on various waxes such as beeswax or carnauba (Chiumarelli & Hubinger, 2012; Valencia‐Chamorro et al., 2009; Zhao, Cao, & Zhu, 2011). In this study, four different carnauba wax‐based coatings including wax embedded with 1% OPEO, incorporated with 2% MMT and combination of 0.5% OPEO and 1% MMT was used for blood citrus.

The total phenol content of samples showed no differences among different wax treatments. However, phenolic content increased at the end of the storage. This could be related to the polyphenol oxidase activity causing the oxidation of phenols and turning them into quinons (Stanley, 1998). Furthermore, aging process might be affected on total phenolic reduction (Scalzo, Iannoccari, Summa, Morelli, & Rapisarda, 2004). Generally, the polyphenols content in citrus fruits depend on the storage conditions such as temperature and length of storage (Klimczak, Małecka, Szlachta, & Gliszczyńska‐Świgło,^2007^). The amount of phenols may be increased or decreased after harvest based on the type of fruits and vegetables that is related to storage conditions (Singleton, Orthofer, & Lamuela‐Raventós, 1999). The similar results were explained by Klimczak et al. (2007) and verified that time of storage and temperature have strong influence on total phenol. The amount of total phenols reduced in wax coating and wax‐free fruits which was according to the results of Lim, Lim, and Tee (2006) about mango. However, Rapisarda, Bianco, Pannuzzo, and Timpanaroɴs (2008) studies indicated that the amount of anthocyanins, flavanol, and hydroxycinnamic acid increased during storage, but vitamin C was reduced.

The antioxidant activity of fruits juice was expressed by the DPPH radical scavenging assay. Antioxidants including vitamin C, polyphenols, and carotenoids are useful in diets for the prevention of diseases (Kris‐Etherton et al., 2002). In spite of all that, the alteration of flavonoid content, ascorbic acid, and antioxidant capacity in different fruits such as oranges, tangerines, and grapefruit reported was attributed to the reduction of antioxidant capacity during the storage (Gardner, White, McPhail, & Duthie, 2000). Thus, coating as an appropriate approach can be used to protect the antioxidant components. According to the results, antioxidant activity of fruit juice increased throughout the storage time. This could be conformable with results of the total phenolic content and exhibited the protective effect of wax coating as well as incorporation of nanoclays. In this manner, MMT played an important role in barrier properties.

In addition, the amount of anthocyanin, flavanols, and hydroxy cinnamic acid in the blood orange usually increases during the storage (Rapisarda et al., 2008). So high antioxidant activity of blood oranges during storage could be due to the synthesis of phenolic compounds as mentioned above although it can be attributed to the species. However, the temperature and storage time should be considered (Klimczak et al., 2007).

The results showed that total soluble solids gradually increased during storage which might be due to sugar synthesis from organic acids (Rapisarda et al., 2008). Degradation of cell wall may also lead to increase in total dissolved solids (Burns, 1990). Nonetheless, total soluble solids during storage have the uptrend as a result of hydrolytic enzymes activity or waste water under storage conditions (Dris, Niskanen, & Jain, 2003). The results of this study were conformable with the study of Shahid and Abbasi (2011) which reported that the increase in soluble solids of orange over storage and lower amount was observed in fruits beeswax and cellulose coated.

In citrus, the predominant form of vitamin C is ascorbic acid, whereas dehydroascorbic acid (DHA) is less than 10% of total vitamin C content. Moreover, the changes in DHA values during the storage are negligible; thus, the amount of ascorbic acid can be assumed as vitamin C content in juice (Rapisarda et al., 2008). The results of vitamin C indicated the reduction trend in all samples; however, those coated with wax reinforced with MMT and MMT + OPEO showed higher vitamin C content. The reduction of ascorbic acid during the storage might be related to the increasing in respiration rate because vitamin C is sensitive to oxidation deterioration (Hassan, Lesmayati, Qomariah, & Hasbianto, 2014). It seems control had higher respiration and higher vitamin C loss. In contrast, fruits treated with wax containing MMT had higher vitamin C content. This results are attributed to excellent oxygen barrier properties of reinforced coatings with MMT which reduce the rate of vitamin C oxidation (Azeredo et al., 2012; Bendahou, Kaddami, Espuche, Gouanvé, & Dufresne, 2011; Sanchez‐Garcia & Lagaron, 2010). However, the amount of vitamin C in the fruit is influenced by storage time and temperature (Klimczak et al., 2007). The same observation was gained from Azeredo et al. (2012) who claimed that nanocomposite coating (MMT and nano‐cellulose) enhanced further maintenance of vitamin C.

In addition, Arena, Fallico, and Maccarone (2001) observed the reduction of vitamin C during the long time storage in commercial oranges.

The results of titratable acidity showed a reduction trend in all fruits coated during the storage; however, the wax coatings incorporated with MMT had higher acidity rather than others. It seems that MMTs play an important role in maintenance of acidity. Titratable acidity is an important quality indicator for citrus fruit ripening and directly related to the concentration of organic acids such as citric acid and malic acid. This reduction of acidity might be related to the consumption of the malic and citric acid during ripening (Shahid & Abbasi, 2011) or used as source of carbon for alcoholic fermentation and even synthesis of polyphenols such as anthocyanins and non anthocyanins phenols (Rapisarda et al., 2008). In addition, coating acts as barrier to gas permeation which causes the accumulation of CO_2_ and motivate of anaerobic respiration (Arnon, Zaitsev, Porat, & Poverenov, 2014). Furthermore, MMT clays disperse in polymer coating matrix and create a maze path that causes slowly gases permeation rate (Kim & Cha, 2014). The similar results were gained by Hassan et al. (2014) who examined the effect of bees wax coating on quality of tangerine citrus. They obtained the decline in titratable acidity; however, there were no significant difference. Also, Zeng, Zhang, Chen, and Fu (2012) found that the amount of titratable acidity decreases over time and oranges coated by clove bud extracts had higher acidity than control. Thus, it seems low temperature storage and the use of appropriate coating is necessary for maintaining and increasing biologic properties citrus postharvest (Rapisarda et al., 2008). There are numerous researches that verified the reduction of titratable acidity for other fruits (Dris et al., 2003; Obenland, Collin, Mackey, Sievert, & Arpaia, 2011; Robards, Li, Antolovich, & Boyd, 1997; Tietel et al., 2010).

The results of firmness revealed that the wax + MMT coating maintained the appropriate firmness of fruits while wax‐coating‐treated fruits and control had the lowest stiffness. Water loss during the storage causes rapid deterioration by shriveling. Maintaining the quality of fruit could be achieved by reducing water loss from fruit during storage (Chien et al., 2007). In this manner, edible coatings can be stated as a barrier against water loss, thereby preventing the reduction of cell pressure and of the cell membrane break which prevent the reduction of the firmness (Bartolozzo, Borneo, & Aguirre, 2016). In addition, coating supplemented with MMT clay nanomaterials got reinforced and as nanofillers improved the water vapor barrier properties (Azeredo et al., 2012). The results of this study are in agreement with Hashemi and Taghinezhad (2012) who evaluated the effect of nanocomposites (nanoclay–chitosan) on lemon. They claimed coating had significant effect on firmness which verified our results. Moreover, according to the results of the Njombolwana et al. (2013), carnauba coating showed the low firmness loss ratio (0.74) on sweet orange fruit as our experimental data exhibited. In addition, there are various studies about coating effects on quality and firmness. For instance, the same results were observed about of chitosan for strawberry fruit (Del‐Valle, Hernández‐Muñoz, Guarda, & Galotto, 2005). Also, the study on the Persian lime wax coating indicated that the use of wax causes greater uniformity, increased stiffness, and reduced defects treated fruits (Chien et al., 2007). The results of Valencia‐Chamorro et al. (2009) about the effect of edible coatings on Valencia orange showed that the firmness and inside gas concentrations were affected. Moreover, Shahid and Abbasi (2011) evaluated the effect of bee wax coating treatments on the firmness (kg/cm^2^) of sweet orange cv. “Blood Red” during 2004 and 2005. The results showed the application of 5% bee wax in 2005 retained maximum firmness (5.13 kg/cm^2^) throughout the storage period.

Color is one of the most important factors in admission of fruit quality. Edible coatings based on natural polymers are capable of delaying the extrinsic fruit and vegetables color and improve appearance of the product (Azeredo et al., 2012). According to the results, coatings containing MMT presented “L” and “b” values are similar to others. While the samples coated with MMT showed the highest “a” value throughout the entire storage period, whereas the pure wax coating slightly increased the sample redness due to the fact that the natural color of the nanoclay is more clearly expressed in this coating. Furthermore, MMT played an important role in stability of anthocyanin and protected it against the visible light led to maintain the citrus original color (Kohno, Hoshino, Matsushima, Tomita, & Kobayashi, 2007; Lima, Martinez‐Ortiz, Fregoso, & Mendez‐Vivar, 2007). These results had been confirmed by Azeredo et al. (2012) who described the correlation between MMT effect and maintenance of acerola puree color. It seems the electrostatic field between clay nanoparticles impact on dye stabilization. The study about coating of Acerola Berry based on alginate–acerola including the MMT and nano‐cellulose indicated that MMT‐coated samples were preferred rather than others because they had the color properties as same as fresh Acerola (Azeredo et al., 2012).

The results of pH were conformed with titratable acidity results and as predicted an increase in pH was observed. The change in pH during whole storage period might be due to number of reasons; first, the alteration of biochemical condition of fruit due to treatments and second, due to lower rate of respiration and metabolic activity. pH value increases but at a slower rate particularly at the end of storage period, as there might be the saturation of atmosphere inside the pack with water vapors (Biasi & Zanette, 2000; Shahid & Abbasi, 2011). In addition, reduction of pH during storage could be related to oxidation of food components such as aldehydes and ketones as well as high CO_2_ concentration because of the oxygen and water vapor barrier properties of coatings, especially MMT impact on accumulation of CO_2_ and increasing the pH (Manzano & Diaz, 2001; Pesis & Ben‐Arie, 1986).

Our results are in agreement with finding of Hashemi and Taghinezhad (2012) which evaluated the chitosan nanocomposite coating (chitosan–clay) on the lemon quality.

## 5 CONCLUSION

In this study, the physicochemical properties, maintenance of quality and the extension of shelf life of blood orange by coating are demonstrated.

Fruits were coating based on carnauba wax with MMT (2%), carnauba with OPEO (1%), carnauba combination with MMT (1%) and OPEO (0.5%), and coating based on carnauba wax alone. The results showed that coating had significant effect on decay and no trace of decay was observed. Also wax coating had maximum deformation which was same as control and minimum amount was related to MMT that had no significant difference with orange peel essential oil and MMT + OPEO. Furthermore, the carnauba + MMT coating, respectively, had the maximum and minimum amount of total acidity and dissolved solid. In addition, time and coating had significant effect on vitamin C in which maximum and minimum amount was observed in MMT and combination of MMT + OPEO treatments, respectively. Total phenol analysis of coated samples had a difference with control. Moreover, DPPH evaluation showed the highest antioxidant activity of MMT coating. In addition, component “L” and “a” had the maximum changes in OPEO treatment and MMT coating, respectively.

To conclude, the incorporation of MMT into the carnauba wax coating is an alternative approach for elongation of fruit shelf life like blood range while preserving their freshness.

## CONFLICT OF INTEREST

None declared.

## ETHICAL REVIEW

This study involved no human or animal testing.

## References

[fsn3681-bib-0001] AOAC (2005). Official method of analyses. Association of Official Analytical Chemists (No. 934.06).

[fsn3681-bib-0002] Arena, E. , Fallico, B. , & Maccarone, E. (2001). Evaluation of antioxidant capacity of blood orange juices as influenced by constituents, concentration process and storage. Food Chemistry, 74, 423–427. 10.1016/S0308-8146(01)00125-X

[fsn3681-bib-0003] Arnon, H. , Zaitsev, Y. , Porat, R. , & Poverenov, E. (2014). Effects of carboxymethyl cellulose and chitosan bilayer edible coating on postharvest quality of citrus fruit. Postharvest Biology and Technology, 87, 21–26. 10.1016/j.postharvbio.2013.08.007

[fsn3681-bib-0004] Azeredo, H. M. , Miranda, K. W. , Ribeiro, H. L. , Rosa, M. F. , & Nascimento, D. M. (2012). Nanoreinforced alginate–acerola puree coatings on acerola fruits. Journal of Food Engineering, 113, 505–510. 10.1016/j.jfoodeng.2012.08.006

[fsn3681-bib-0005] Bartolozzo, J. , Borneo, R. , & Aguirre, A. (2016). Effect of triticale‐based edible coating on muffin quality maintenance during storage. Journal of Food Measurement and Characterization, 10, 88–95. 10.1007/s11694-015-9280-1

[fsn3681-bib-0006] Bendahou, A. , Kaddami, H. , Espuche, E. , Gouanvé, F. , & Dufresne, A. (2011). Synergism effect of montmorillonite and cellulose whiskers on the mechanical and barrier properties of natural rubber composites. Macromolecular Materials and Engineering, 296, 760–769. 10.1002/mame.201000444

[fsn3681-bib-0007] Biasi, L. A. , & Zanette, F. (2000). Gibberellic acid alone or associated with wax in the post‐harvest of “Tahiti” lime. Scientia‐Agraria, 1(1, 2), 39–44. 10.5380/rsa.v1i1.966

[fsn3681-bib-0008] Bidchol, A. M. , Wilfred, A. , Abhijna, P. , & Harish, R. (2011). Free radical scavenging activity of aqueous and ethanolic extract of *Brassica oleracea* L. var. italica. Food and Bioprocess Technology, 4, 1137–43. 10.1007/s11947-009-0196-9

[fsn3681-bib-0009] Boelter, J. F. , & Brandelli, A. (2016). Innovative bionanocomposite films of edible proteins containing liposome‐encapsulated nisin and halloysite nanoclay. Colloids and Surfaces B: Biointerfaces, 145, 740–747. 10.1016/j.colsurfb.2016.05.080 27289315

[fsn3681-bib-0010] Bonina, F. , Leotta, C. , Scalia, G. , Puglia, C. , Trombetta, D. , Tringali, G. , … Saija, A. (2002). Evaluation of oxidative stress in diabetic patients after supplementation with a standardised red orange extract. Diabetes, Nutrition & Metabolism, 15, 14–19. 11942734

[fsn3681-bib-0011] Bourtoom, T. (2008). Edible films and coatings: Characteristics and properties. International Food Research Journal, 15, 237–248.

[fsn3681-bib-0012] Burns, J. K. (1990). α‐and β‐galactosidase activities in juice vesicles of stored Valencia oranges. Phytochemistry, 29, 2425–2429. 10.1016/0031-9422(90)85160-H

[fsn3681-bib-0013] Chien, P.‐J. , Sheu, F. , & Lin, H.‐R. (2007). Coating citrus (*Murcott tangor*) fruit with low molecular weight chitosan increases postharvest quality and shelf life. Food Chemistry, 100, 1160–1164. 10.1016/j.foodchem.2005.10.068

[fsn3681-bib-0014] Chiumarelli, M. , & Hubinger, M. D. (2012). Stability, solubility, mechanical and barrier properties of cassava starch–Carnauba wax edible coatings to preserve fresh‐cut apples. Food Hydrocolloids, 28, 59–67. 10.1016/j.foodhyd.2011.12.006

[fsn3681-bib-0015] Colla, E. , Do Amaral Sobral, P. J. , & Menegalli, F. C. (2006). Amaranthus cruentus flour edible films: Influence of stearic acid addition, plasticizer concentration, and emulsion stirring speed on water vapor permeability and mechanical properties. Journal of Agricultural and Food Chemistry, 54, 6645–6653. 10.1021/jf0611217 16939322

[fsn3681-bib-0016] Danalache, F. , Carvalho, C. Y. , Alves, V. D. , Moldão‐Martins, M. , & Mata, P. (2016). Optimisation of gellan gum edible coating for ready‐to‐eat mango (*Mangifera indica* L.) bars. International Journal of Biological Macromolecules, 84, 43–53. 10.1016/j.ijbiomac.2015.11.079 26657585

[fsn3681-bib-0017] Davies, K. M. (2007). Genetic modification of plant metabolism for human health benefits. Mutation Research/Fundamental and Molecular Mechanisms of Mutagenesis, 622, 122–137. 10.1016/j.mrfmmm.2007.02.003 17382356

[fsn3681-bib-0018] De Pascual‐Teresa, S. , Moreno, D. A. , & García‐Viguera, C. (2010). Flavanols and anthocyanins in cardiovascular health: A review of current evidence. International Journal of Molecular Sciences, 11, 1679–1703. 10.3390/ijms11041679 20480037 PMC2871133

[fsn3681-bib-0019] Del‐Valle, V. , Hernández‐Muñoz, P. , Guarda, A. , & Galotto, M. (2005). Development of a cactus‐mucilage edible coating (*Opuntia ficus indica*) and its application to extend strawberry (*Fragaria ananassa*) shelf‐life. Food Chemistry, 91, 751–756. 10.1016/j.foodchem.2004.07.002

[fsn3681-bib-0021] Dris, R. , Niskanen, R. , & Jain, S. M. (2003). Crop management and postharvest handling of horticultural products. Volume II: Fruits and vegetables. Enfield, USA: Science Publishers Inc.

[fsn3681-bib-0022] Echegoyen, Y. (2015). Nano‐developments for food packaging and labeling applications In Rai M., Ribeiro C., Mattoso L., & Duran N. (Eds.), Nanotechnologies in food and agriculture (pp. 141–166). New York, NY: Springer.

[fsn3681-bib-0023] Egan, H. , Kirk, R. S. , & Sawyer, R. (1981). Pearson's chemical analysis of foods, 8th ed. London, UK: Churchill Livingstone Ltd..

[fsn3681-bib-0024] Forato, L. A. , de Britto, D. , de Rizzo, J. S. , Gastaldi, T. A. , & Assis, O. B. (2015). Effect of cashew gum‐carboxymethylcellulose edible coatings in extending the shelf‐life of fresh and cut guavas. Food Packaging and Shelf Life, 5, 68–74. 10.1016/j.fpsl.2015.06.001

[fsn3681-bib-0025] Gardner, P. T. , White, T. A. , McPhail, D. B. , & Duthie, G. G. (2000). The relative contributions of vitamin C, carotenoids and phenolics to the antioxidant potential of fruit juices. Food Chemistry, 68, 471–474. 10.1016/S0308-8146(99)00225-3

[fsn3681-bib-0026] Guerreiro, A. C. , Gago, C. M. , Faleiro, M. L. , Miguel, M. G. , & Antunes, M. D. (2017). The effect of edible coatings on the nutritional quality of ‘Bravo de Esmolfe'fresh‐cut apple through shelf‐life. LWT‐Food Science and Technology, 75, 210–219. 10.1016/j.lwt.2016.08.052

[fsn3681-bib-0027] Hashemi, J. , & Taghinezhad, E. (2012). Effects of nano composite coating on the lemon quality. International Conference of Agricultural Engineering, Valencia Conference Center.

[fsn3681-bib-0028] Hassan, Z. H. , Lesmayati, S. , Qomariah, R. , & Hasbianto, A. (2014). Effects of wax coating applications and storage temperatures on the quality of tangerine citrus (*Citrus reticulata*) var. Siam Banjar. International Food Research Journal, 21(2), 641–648.

[fsn3681-bib-0029] Hernández‐Muñoz, P. , Almenar, E. , Ocio, M. J. , & Gavara, R. (2006). Effect of calcium dips and chitosan coatings on postharvest life of strawberries (Fragaria x ananassa). Postharvest Biology and Technology, 39, 247–253. 10.1016/j.postharvbio.2005.11.006

[fsn3681-bib-0031] Kelebek, H. , Canbas, A. , & Selli, S. (2008). Determination of phenolic composition and antioxidant capacity of blood orange juices obtained from cvs. Moro and Sanguinello (*Citrus sinensis* (L.) Osbeck) grown in Turkey. Food Chemistry, 107, 1710–1716. 10.1016/j.foodchem.2007.10.004

[fsn3681-bib-0032] Kim, S. W. , & Cha, S. H. (2014). Thermal, mechanical, and gas barrier properties of ethylene–vinyl alcohol copolymer‐based nanocomposites for food packaging films: Effects of nanoclay loading. Journal of Applied Polymer Science, 131(11).

[fsn3681-bib-0033] Klimczak, I. , Małecka, M. , Szlachta, M. , & Gliszczyńska‐Świgło, A. (2007). Effect of storage on the content of polyphenols, vitamin C and the antioxidant activity of orange juices. Journal of Food Composition and Analysis, 20, 313–322. 10.1016/j.jfca.2006.02.012

[fsn3681-bib-0034] Kohno, Y. , Hoshino, R. , Matsushima, R. , Tomita, Y. , & Kobayashi, K. (2007). Stabilization of flavylium dyes by incorporation in the clay interlayer. Shikizai kyokaishi, 80, 6–12.

[fsn3681-bib-0035] Koleva, I. I. , van Beek, T. A. , Linssen, J. P. , Groot, A. D. , & Evstatieva, L. N. (2002). Screening of plant extracts for antioxidant activity: A comparative study on three testing methods. Phytochemical Analysis, 13, 8–17. 10.1002/(ISSN)1099-1565 11899609

[fsn3681-bib-0036] Kris‐Etherton, P. M. , Hecker, K. D. , Bonanome, A. , Coval, S. M. , Binkoski, A. E. , Hilpert, K. F. , … Etherton, T. D. (2002). Bioactive compounds in foods: Their role in the prevention of cardiovascular disease and cancer. The American Journal of Medicine, 113, 71–88. 10.1016/S0002-9343(01)00995-0 12566142

[fsn3681-bib-0037] Lester, G. E. , & Hodges, D. M. (2008). Antioxidants associated with fruit senescence and human health: Novel orange‐fleshed non‐netted honey dew melon genotype comparisons following different seasonal productions and cold storage durations. Postharvest Biology and Technology, 48, 347–354. 10.1016/j.postharvbio.2007.11.008

[fsn3681-bib-0038] Lim, Y. Y. , Lim, T. T. , & Tee, J. J. (2006). Antioxidant properties of guava fruit: Comparison with some local fruits. Sunway Academic Journal, 3, 9–20.

[fsn3681-bib-0039] Lima, E. , Martinez‐Ortiz, M. , Fregoso, E. , & Mendez‐Vivar, J. (2007). Capturing natural chromophores on natural and synthetic aluminosilicates. Studies in Surface Science and Catalysis, 170, 2110–2115. 10.1016/S0167-2991(07)81107-4

[fsn3681-bib-0040] Manzano, J. , & Diaz, A. (2001). Effect of storage time, temperature and wax coating on the quality of fruits of ‘Valencia'orange (*Citrus sinensis* L.). Proceedings of the Interamerican Society for Tropical Horticulture, 44, 24–29.

[fsn3681-bib-0041] Martín‐Belloso, O. , Soliva‐Fortuny, R. , & Baldwin, E. (2005). Conservación mediante recubrimientos comestibles *Nuevas tecnologías de conservación de productos vegetales frescos cortados* Primera Edición (pp. 341–356). Guadalajara, México: Logiprint Digital S.

[fsn3681-bib-0042] McDonald, S. , Prenzler, P. D. , Antolovich, M. , & Robards, K. (2001). Phenolic content and antioxidant activity of olive extracts. Food Chemistry, 73, 73–84. 10.1016/S0308-8146(00)00288-0

[fsn3681-bib-0043] Njombolwana, N. S. , Erasmus, A. , van Zyl, J. G. , du Plooy, W. , Cronje, P. J. , & Fourie, P. H. (2013). Effects of citrus wax coating and brush type on imazalil residue loading, green mould control and fruit quality retention of sweet oranges. Postharvest Biology and Technology, 86, 362–371. 10.1016/j.postharvbio.2013.07.017

[fsn3681-bib-0044] Obenland, D. , Collin, S. , Mackey, B. , Sievert, J. , & Arpaia, M. L. (2011). Storage temperature and time influences sensory quality of mandarins by altering soluble solids, acidity and aroma volatile composition. Postharvest Biology and Technology, 59(2), 187–193. 10.1016/j.postharvbio.2010.09.011

[fsn3681-bib-0045] Paredes‐López, O. , Cervantes‐Ceja, M. L. , Vigna‐Pérez, M. , & Hernández‐Pérez, T. (2010). Berries: Improving human health and healthy aging, and promoting quality life—a review. Plant Foods for Human Nutrition, 65, 299–308. 10.1007/s11130-010-0177-1 20645129

[fsn3681-bib-0046] Pesis, E. , & Ben‐Arie, R. (1986). Carbon dioxide assimilation during postharvest removal of astringency from persimmon fruit. Physiologia Plantarum, 67, 644–648. 10.1111/j.1399-3054.1986.tb05071.x

[fsn3681-bib-0047] Rapisarda, P. , Bianco, M. L. , Pannuzzo, P. , & Timpanaro, N. (2008). Effect of cold storage on vitamin C, phenolics and antioxidant activity of five orange genotypes [*Citrus sinensis* (L.) Osbeck]. Postharvest Biology and Technology, 49, 348–354. 10.1016/j.postharvbio.2008.02.002

[fsn3681-bib-0048] Ray, S. S. , & Okamoto, M. (2003). Polymer/layered silicate nanocomposites: A review from preparation to processing. Progress in Polymer Science, 28, 1539–1641.

[fsn3681-bib-0049] Robards, K. , Li, X. , Antolovich, M. , & Boyd, S. (1997). Characterisation of citrus by chromatographic analysis of flavonoids. Journal of the Science of Food and Agriculture, 75, 87–101. 10.1002/(ISSN)1097-0010

[fsn3681-bib-0050] Rojas‐Argudo, C. , del Río, M. , & Pérez‐Gago, M. (2009). Development and optimization of locust bean gum (LBG)‐based edible coatings for postharvest storage of ‘Fortune' mandarins. Postharvest Biology and Technology, 52, 227–234. 10.1016/j.postharvbio.2008.11.005

[fsn3681-bib-0051] Sanchez‐Garcia, M. D. , & Lagaron, J. M. (2010). On the use of plant cellulose nanowhiskers to enhance the barrier properties of polylactic acid. Cellulose, 17, 987–1004. 10.1007/s10570-010-9430-x

[fsn3681-bib-0052] Scalzo, R. L. , Iannoccari, T. , Summa, C. , Morelli, R. , & Rapisarda, P. (2004). Effect of thermal treatments on antioxidant and antiradical activity of blood orange juice. Food Chemistry, 85, 41–47. 10.1016/j.foodchem.2003.05.005

[fsn3681-bib-0053] Scordino, M. , Sabatino, L. , Lazzaro, F. , Borzì, M. A. , Gargano, M. , Traulo, P. , & Gagliano, G. (2015). Blood orange anthocyanins in fruit beverages: How the commercial shelf life reflects the quality parameter. Beverages, 1, 82–94. 10.3390/beverages1020082

[fsn3681-bib-0054] Selli, S. , & Kelebek, H. (2011). Aromatic profile and odour‐activity value of blood orange juices obtained from Moro and Sanguinello (*Citrus sinensis* L. Osbeck). Industrial Crops and Products, 33, 727–733. 10.1016/j.indcrop.2011.01.016

[fsn3681-bib-0055] Shahid, M. N. , & Abbasi, N. A. (2011). Effect of bee wax coatings on physiological changes in fruits of sweet orange CV “blood red”. Sarhad Journal of Agriculture, 27, 385–394.

[fsn3681-bib-0056] Shokrieh, M. M. , Saeedi, A. , & Chitsazzadeh, M. (2013). Mechanical properties of multi‐walled carbon nanotube/polyester nanocomposites. Journal of Nanostructure in Chemistry, 3, 20 10.1186/2193-8865-3-20

[fsn3681-bib-0057] Singleton, V. L. , Orthofer, R. , & Lamuela‐Raventós, R. M. (1999). [14] Analysis of total phenols and other oxidation substrates and antioxidants by means of folin‐ciocalteu reagent. Methods in Enzymology, 299, 152–178. 10.1016/S0076-6879(99)99017-1

[fsn3681-bib-0058] Sorrentino, A. , Gorrasi, G. , & Vittoria, V. (2007). Potential perspectives of bio‐nanocomposites for food packaging applications. Trends in Food Science & Technology, 18, 84–95. 10.1016/j.tifs.2006.09.004

[fsn3681-bib-0059] Stanley, J. (1998). Postharvest physiology of perishable plant products (pp. 143–256). New Delhi, India: CSB Publ. Distrib.

[fsn3681-bib-0060] Talens, P. , & Krochta, J. M. (2005). Plasticizing effects of beeswax and carnauba wax on tensile and water vapor permeability properties of whey protein films. Journal of Food Science, 70, E239–E243.

[fsn3681-bib-0061] Tietel, Z. , Bar, E. , Lewinsohn, E. , Feldmesser, E. , Fallik, E. , & Porat, R. (2010). Effects of wax coatings and postharvest storage on sensory quality and aroma volatile composition of ‘Mor' mandarins. Journal of the Science of Food and Agriculture, 90(6), 995–1007. 20355140 10.1002/jsfa.3909

[fsn3681-bib-0063] Valencia‐Chamorro, S. A. , Pérez‐Gago, M. B. , del Río, M. Á. , & Palou, L. (2009). Effect of antifungal hydroxypropyl methylcellulose (HPMC)–lipid edible composite coatings on postharvest decay development and quality attributes of cold‐stored ‘Valencia' oranges. Postharvest Biology and Technology, 54, 72–79. 10.1016/j.postharvbio.2009.06.001

[fsn3681-bib-0064] Vargas, M. , Pastor, C. , Chiralt, A. , McClements, D. J. , & Gonzalez‐Martinez, C. (2008). Recent advances in edible coatings for fresh and minimally processed fruits. Critical Reviews in Food Science and Nutrition, 48, 496–511. 10.1080/10408390701537344 18568856

[fsn3681-bib-0065] Wardowski, W. M. , Hall, D. J. , & Grierson, W. (2006). Fresh citrus fruits (2nd ed., pp. 547–581). Ocala, FL: Florida Science Source Inc..

[fsn3681-bib-0066] Yaman, Ö. , & Bayoιndιrlι, L. (2002). Effects of an edible coating and cold storage on shelf‐life and quality of cherries. LWT‐Food science and Technology, 35, 146–150. 10.1006/fstl.2001.0827

[fsn3681-bib-0067] Zeng, R. , Zhang, A. , Chen, J. , & Fu, Y. (2012). Postharvest quality and physiological responses of clove bud extract dip on ‘Newhall' navel orange. Scientia Horticulturae, 138, 253–258. 10.1016/j.scienta.2012.02.036

[fsn3681-bib-0068] Zhao, K. , Cao, X. D. , & Zhu, S. X. (2011). Effects of different concentrations of beeswax coating agent on Taiwan green jujube during storage. Storage & Process, 4, 7.

